# Effect of foot reflexology on relieving pain and improving resilience among patients undergoing coronary artery bypass graft

**DOI:** 10.1186/s12912-025-03860-w

**Published:** 2025-09-10

**Authors:** Ayman Muhammad Kamel Senosy, Rasha Hassan Abbas Shady, Zeinab Adham Ahmed, Amina Abdelrazek Aldeeb, Manal Saleh Moustafa, Tabasem Fayez Fatah-Allah Hegazy

**Affiliations:** 1 Medical-Surgical Nursing, Faculty of Nursing, Ain Shams University, Cairo, Egypt; 2 Medical-Surgical Nursing, Faculty of Nursing, Galala University, Suez, Egypt; 3 Medical-Surgical Nursing, Faculty of Nursing, Mansoura University, Mansoura, Egypt; 4 Medical-Surgical Nursing, Faculty of Nursing, October 6 University, Cairo, Egypt; 5 Obstetric Nursing, Faculty of Nursing, Galala University, Suez, Egypt; 6 College of Applied Medical Science, Shaqra University, Shaqra, Saudi Arabia; 7 Faculty of Nursing, Zagazig University, Zagazig, Egypt; 8 Lecturer of Faculty of Psychiatric and Mental Health Nursing, Ain Shams University, Cairo, Egypt

**Keywords:** Nursing program, Foot reflexology, Nursing interventions, CABG

## Abstract

**Background:**

By applying pressure to specific reflective areas on the foot in accordance with different organs, standardized nursing interventions and programs significantly reduce pain. Increased intervention time can improve the effectiveness of foot reflexology, which is regarded as a beneficial intervention in a patient care program. It has been shown to have many health benefits, including lowering pain levels.

**Aim:**

This study aimed to explore the effect of foot reflexology on relieving pain and improving resilience among patients undergoing coronary artery bypass graft.

**Methods:**

A quasi-experimental (one group pre- and posttest) to evaluate the effectiveness of foot reflexology intervention on relieving pain and improving resilience among patients undergoing CABG. Setting: The study has been conducted in the intensive care unit and intermediate care units at the Academic Institute for Cardiothoracic Surgery, which is affiliated with Ain Shams University Hospitals. A purposive sample of patients (N.50) with coronary artery bypass graft surgery (CABG) has been recruited for the conduction of the present study. The study period was 9 months, from April 2024 to December 2024. Data collection tools: (1) structured interview questionnaire, (2) visual analog scale (VAS), and (3) the Connor–Davidson Resilience Scale (CD-RISC).

**Results:**

In terms of age, 72% of the patients were male, and the mean ± SD was 40.20 ± 7.64. Regarding the visual analog scale (VAS) “Pain Scale,” the study found that, with a p-value of *p* < 0.001, there was a statistically significant decrease in pain post the intervention as opposed to pre. A p-value of *p* < 0.001 indicated that the mean resilience value was statistically significant higher in the post-intervention period than in the pre-intervention period. With a p-value of *p* < 0.001, there was a statistically significant decrease in pain post the intervention as compared to pre.

**Conclusion:**

The results of the present investigation indicate that the use of foot reflexology following open cardiac surgery greatly relieves the pain. Foot reflexology showed a more notable and vital improvement after the coronary artery bypass graft for patients as nonpharmacological treatment for patients as nursing care. Recommendations: Application of a foot reflexology training program for nurses who work in open heart surgery units so they may incorporate it into their regular patient care. Patients should follow foot reflexology training as part of their routine care after open heart surgery. Provide nursing personnel with regular in-service training to aid in the appropriate management of CABG patients to enhance their quality of life and results.

**Clinical trial number:**

Not applicable

**Supplementary Information:**

The online version contains supplementary material available at 10.1186/s12912-025-03860-w.

## Introduction

Globally, cardiovascular illnesses account for most deaths, with coronary artery disease (CAD) emerging as the leading cause of both death and disability. CABG surgery is used to treat CAD patients when medicinal interventions are insufficient to restore the coronary blood flow. Numerous postoperative issues, including anxiety, sadness, discomfort, self-care, and a lower quality of life, are linked to CABG surgery. Following CABG surgery, patients reported feeling less anxious, which was linked to a higher quality of life [1].

By constructing a bypass on obstructed coronary arteries, Coronary Artery Bypass Surgery (CABG) is an interventional treatment used to restore cardiac circulation. CABG is regarded as a convincing therapy to enhance the physical and mental well-being of individuals with cardiovascular problems [2]. Most patients experience pain following the CABG operation. While 75% of patients have reported postoperative discomfort, many patients experience postoperative CABG pain. Mild to moderate pain was reported in 67% of patients and severe pain in 7% of patients on the fourth day following the CABG treatment [3].

After coronary artery bypass graft surgery, nurses can safely use reflexology as a non-pharmacological, non-surgical, and economical way to manage pain and anxiety [4]. Numerous research concluded that reflexology was a useful and complementary non-pharmacological pain management technique for a range of patient groups. Therefore, it is suggested that reflexology, a complementary therapy technique that any nurse can use on their own, would help patients who underwent coronary artery bypass graft surgery experience less pain following the procedure, as well as experience less anxiety and more resilience [5].

Consequently, it is crucial to comprehend While waiting for heart surgery, patients frequently deal with psychological stressors such as anxiety, melancholy, uncertainty, and worry. Following surgery, these factors may lead to a poor prognosis for recovery. In addition to affecting the patient’s degree of satisfaction, pain can lengthen the length of time spent in the hospital following surgery. Following major heart surgery, patients frequently experience extreme pain, tension, and anxiety, which can negatively impact their quality of life, recuperation, and course of treatment [6].

Resilience is the ability to adapt to life’s difficulties and hazards and to act and be effective in stressful situations rather than react and remain inactive. In addition to being dynamic and adaptable in the face of adversity, resilient individuals are better able to adjust to life’s changes and develop and broaden a repertoire of coping mechanisms that help them get through difficult times [7].

Resilience is the process that helps individuals build emotional qualities to deal well with the obstacles in family and social life. Since resilience is not a preset ability, there is no one right approach to learn it. A person’s acquired set of abilities, values, and attitudes are all part of their resilience. Self-awareness, coping with despair and bad moods, anger control, stress management, and problem-solving abilities are some of the foundational abilities for developing resilience [8].

Foot reflexology significantly reduced the increase in pain and anxiety experienced by patients undergoing coronary artery bypass graft surgery, particularly after the removal of the chest tube. Foot reflexology has been shown to reduce discomfort and increase resilience in individuals undergoing coronary artery bypass graft (CABG) surgery. According to research, it can lessen the intensity of pain and anxiety while also improving physiological indicators like oxygen saturation. Reflexology can be easily incorporated into post-operative treatment as a non-invasive supplemental therapy [9]. Stress management training, which combines several forms of relaxation, imagery, effective coping training, self-expression, and anger control, is one way to increase resilience [10].

To ensure the success of nursing procedures, nurses perform a vital and effective role in providing counseling, guidance, and advice. The patients have had less pain, worry, anxiety, and discomfort thanks to the nurses. Because foot reflexology massage is straightforward, affordable, and easy to apply, nurses can employ non-pharmacological and alternative therapies to manage injection pain and anxiety. It is also among the most important complementary therapies that nurses use as a nursing intervention. Furthermore, it improves the patient’s capacity for adaptation and is simple enough for older kids to utilize [11].

For patients undergoing coronary artery bypass graft surgery, foot reflexology dramatically decreased the rise in discomfort and anxiety following chest tube removal. When compared to control groups, foot reflexology typically results in lower pain scores. Both just after the operation and in the days that follow the surgery, this pain reduction is evident [9].” As a result, the study investigated how foot reflexology can help patients having coronary artery bypass grafts feel less discomfort and be more resilient.

### Significance of study

Globally, the main cause of death is coronary heart disease (CHD). The World Health Organization (WHO) estimates that 16% of deaths worldwide are caused by CHD. According to data from the Institute of Health Metrics Evaluation (IHME) in 2019, European nations had the highest CHD mortality rate, accounting for about 30% of all deaths, or roughly twice the global average [12].

Furthermore, since 2000, the number of fatalities from CHD has climbed by over 2 million, reaching 8.9 million in 2019. By 2030, this death rate is predicted to rise by an additional 23.3 million individuals. CHD is a major global health issue and a serious health danger. In addition to disability and mortality, this disease can cause biological, psychological, and social issues [13]. To alleviate pain, foot reflexology involves applying pressure on specific reflecting spots on the foot that correspond to different organs, glands, and other body parts. This causes the release of endorphins, which are natural analgesics. Numerous health benefits have been demonstrated by foot reflexology in terms of reducing pain levels [14].

Depressive symptoms and cardiovascular response were lower in those with resilient traits. Thus, building resilience in people helps them to confront the difficulties around them, deal with them actively, and regain their bodily and psychological equilibrium in these circumstances. Conversely, there is a well-established correlation between positive psychological factors and favorable outcomes regarding cardiovascular disease in both heart patients and healthy individuals. Several life skills, including social competence, purposefulness, and realism, are crucial for resilience [8]. Therefore, the purpose of this study was to find out how foot reflexology interventions affected patients undergoing CABG in terms of pain relief and resilience.

### Aim of the study

The study aims to explore the effect of foot reflexology on relieving pain and improving resilience among patients undergoing coronary artery bypass graft through the following two phases:

**Phase one**:

1. Assess patients’ level of pain before implementation program.
2. Assess patients’ level of resilience before program implementation.
3. Develop and implement an educational program for patients based on their needs assessment.
4. Develop and implement foot reflexology intervention for relieving pain and improving resilience among patients undergoing CABG.

**Phase two**:

5. Evaluate the effectiveness of the foot reflexology intervention on relieving pain among patients undergoing CABG.
6. Evaluate the effectiveness of the foot reflexology intervention on improving resilience among patients undergoing CABG.

### Research hypothesis

Foot reflexology intervention:

#### H1

The foot reflexology will relieve pain among patients undergoing CABG.

#### H2

The foot reflexology will improve resilience among patients undergoing CABG.

## Subjects and methods

### Study design

This study is a quasi-experimental design with a one-group pretest-posttest- follow-up approach to explore the effect of foot reflexology on relieving pain and improving resilience among patients undergoing coronary artery bypass graft.

### Study setting

The study has been conducted in the intensive care unit and intermediate care units at the Academic Institute for Cardiothoracic Surgery, which is affiliated with Ain Shams University Hospitals.

### Study subjects

A purposive sample of patients with coronary artery bypass graft surgery (CABG) has been recruited for the conduction of the present study from the previously mentioned settings who meet the following selection inclusion criteria: patients aged more than 20 years old, from both sexes, patients who are free from psychiatric diseases, and did not receive any previous training program regarding foot reflexology.

#### Tools for data collection

Data has been collected by using the following tools: parts of these tools have been developed, and another has been adopted. It has been translated into simplified Arabic by a language expert.

**1. Structured interview questionnaire**: It has been developed by researchers after reviewing related literature [4, 5–11] It is composed of three parts.

- **Part one**: to assess patients’ demographic characteristics such as age, sex, consanguinity, marital status, educational level, occupation, residence, etc.
- **Part two**: to assess the patients’ illness data, including the history of DM, history of HTN, previous open-heart surgery, etc.
- **Part three**: patients’ knowledge regarding foot reflexology.

2. **Visual analog scale (VAS)**: is a pain rating scale first used by [15, 16]. With a single handwritten mark placed at one point along a 10-cm line that depicts a continuum between the two ends of the scale—“no pain” on the left end (0 cm) and the “worst pain” on the right end (10 cm)—scores are based on self-reported measures of symptoms. Measurements from the starting point (left end) of the scale to the patients’ marks are recorded in centimeters and are interpreted as their pain. The values can be used to track pain progression for a patient or to compare pain between patients with similar conditions.

**3. The Connor–Davidson Resilience Scale (CD-RISC).** It was developed by [17], as a means of assessing resilience. The CD-RISC contains 25 items, all of which carry a 5-point range of responses, as follows: not true at all (0), rarely true (1), sometimes true (2), often true (3), and true nearly all the time (4). Connor and Davidson found that these items correspond to five factors. The first factor reflects having high standards, tenacity, and competence “eight items”: (10, 11, 12, 16, 17, 23, 24, & 25). The second factor reflects handling negative emotions, trusting one’s instincts, and perceived benefits of stress “seven items”: (6, 7, 14, 15, 18, 19, & 20). The third factor reflects having a positive attitude to change and secure relationships “five items”: (1, 2, 4, 5, & 8). The fourth one reflects perceived control “three items”: (13, 21, & 22), and the fifth one spirituality “two items”: (3 & 9). The score for the scale is calculated by adding the values of the items. The total score ranges from 100, with higher scores reflecting greater resilience.

### Program for patients

Based on a comprehensive analysis of related literature as well as patients’ needs and problems, program sessions have been designed including objectives, teaching content, timing, photos, instructions, explanations, and examples. The program booklet content has been reviewed by experts in medical-surgical nursing and psychiatric/mental health nursing fields to confirm its safety, validity for covering objectives, accuracy of information, representativeness, and practices used by patients. The program booklet has also been revised for the degree to which studies and theories supported the details of it.

#### Validity & reliability

The standardized tools used in the current study are valid and reliable. The Arabic version has been checked for its relevance, clarity, completeness, simplicity, and applicability.

#### Administrative design

Official approval has been obtained from the dean of the Faculty of Nursing at Ain Shams University; a letter containing the title and the aim of the study has been directed to the director of the previously mentioned setting to obtain approval for data collection.

### Pilot study

To test the tools’ clarity and applicability, the pilot study has been carried out on 10% of the patients studied. There was no alteration, and the tool has been useful. Based on the findings of the pilot study, patients who were part of the research.

### Fieldwork

- The sample has been selected according to the inclusion criteria. The purpose of the study has been simply explained to the patients who will agree to participate in the study prior to any data collection. Data has been collected in the morning and afternoon during the working time of the previously mentioned setting. The study period was 9 months, from April 2024 to December 2024. The 50 participants who consented to take part in the trial were selected to create the group.
- The interested individuals were gathered to constitute the group. The first instrument’s component one, which was taken from the patient records, was used to evaluate the clinical and personal data of the patients.
- The implementation phase took place from June 2024 to September 2024, and the patients’ clinical and personal data were assessed using components of one of the first instruments, which was derived from the patient records. Both the patients’ resilience and their level of pain were monitored twice before the foot reflexology was started (immediately and one hour before the intervention.
- The procedures for doing foot reflexology have been done by the researchers which involved the following steps: First, the patient was positioned supine with a pillow beneath their feet, bending their feet slightly and angling their heads between 30 and 45 degrees. Starting around 10 cm above the patient’s knee, the massaging region was exposed. The researcher first checked the feet to see whether there were any massage barriers before starting to massage. While standing in front of the patient, the researcher started general reflexology by massaging all reflex points in the plantar with the thumb and forefinger, and then specialized reflexology was done through the pressure points of foot reflexes, such as the solar plexus, hypothalamic, pituitary, spinal cord, adrenal gland, and pelvic. First, for the left foot, followed by the right foot (20 min for each). After the foot reflexology massage technique, the physiological parameters of the patients and pain level were recorded twice (immediately and one hour after the intervention). The patients were followed for three consecutive days.

### Statistical methods

- The data collected from the sample studied has been revised, coded, and entered using a personal computer (PC). SPSS for Windows version 25.0 (SPSS, Chicago, IL) was used for all statistical analyses. The mean ± standard deviation (SD) was used to express continuous data that had a normal distribution. Numbers and percentages were used to represent categorical data. A statistical test called a one-way analysis of variance (ANOVA) is used to examine how the means of more than two groups differ from one another. It applied to continuous data to compare more than two variables. To compare variables using categorical data, the chi-square test, which refers to the distribution of a categorical variable in a sample, frequently needs to be compared with the distribution of a categorical variable in another sample. The study has made use of the paired t-test. For the study’s questionnaires, the reliability (internal consistency) test was computed. The threshold for statistical significance was 0.05.

## Results

The results of the study have been presented in tables and figures that are easy to understand and have been analyzed for inferring information, and the proper comments have been stated.

### Statistical analysis

The recorded data were analyzed using the statistical package for social sciences, version 22.0 (SPSS Inc., Chicago, Illinois, USA). Quantitative data were expressed as mean ± standard deviation (SD). Qualitative data were expressed as frequencies and percentages.

The following tests were done:

- *Chi-square (x²)* test of significance was used to compare proportions between qualitative parameters. The chi-square test was used to assess differences in proportions between categorical variables.”
- *A paired sample t-test* of significance was used when comparing between related samples. “The paired sample t-test was used to compare means between related samples.
- *Pearson’s correlation coefficient (r)* test was used to assess the degree of association between two sets of variables. Pearson’s correlation coefficient was used to evaluate the strength and direction of the relationship between two continuous variables.
- The confidence interval was set at 95%, and the margin of error accepted was set to 5%. So, the p-value was considered significant as follows:
- *Probability (P-value)*.
- A p-value *≤* 0.05 was considered significant.
- A p-value *≤* 0.001 was considered highly significant.
- A p-value > 0.05 was considered insignificant.

#### Part I: Socio-demographic data

**Table 1:** Number and percentage distribution of the patients studied according to their socio-demographic data (*N* = 50)

Items	Studied patients (*N* = 50)	
	*N*	%
**Age**		
≤ 29	6	12.0
30-≥49	39	78.0
≥ 50	5	10.0
Mean ± SD	40.20 ± 7.64	
**Gender**		
Male	36	72.0
Female	14	28.0
**Educational Level**		
Illiterate	25	50.0
Read and write	20	40.0
Highly educated	5	10.0
**Marital status**		
Single- Widow- Divorced	13	26.0
Married	37	74.0
**Socioeconomic status**		
Sufficient	18	36.0
Not sufficient	32	64.0
**Treatment cost**		
Free	23	46.0
Self-cost	9	18.0
Medical insurance	18	36.0
**Living area**		
Rural	19	38.0
Urban	31	62.0
**Smoking**		
Yes	29	58.0
No	21	42.0
**Family members smokers**		
Yes	27	54.0
No	23	46.0

Table 1 showed that 78% of the studied patients their age was 30-≥49 and the Mean ± SD was 40.20 ± 7.64, while 72% were males, 64% with insufficient socioeconomic status, and 58% were smokers.

#### Part II: Clinical data sheet (medical health history)

**Table 2:** Number and percentage distribution of the patients studied according to their clinical data sheet (medical health history) (*N* = 50)

Present history	Yes		No	
	No.	%	No.	%
**Have previous None heart surgery**	13	26.0	37	74.0
**Have previous heart surgery**	14	28.0	36	72.0
**Previous history**				
Have family members with heart disease	29	58.0	21	42.0
Have family members with heart surgery	7	14.0	43	86.0
Entering hospital with other disease	32	64.0	18	36.0
Taking medicine for non-heart disease	28	56.0	22	44.0

As regards the clinical data sheet (medical health history), Table 2, revealed that 26% had no previous heart surgeries and only 28% had previous heart surgeries. And 58% and 64% had a family member with heart disease and entering hospital with other disease respectively.

**Table 3:** Number and percentage distribution of the patients studied according to their symptoms and signs suffering, lab investigation and radiological investigations (*N* = 50)

	Yes		No	
	No.	%	No.	%
**Symptoms and signs suffering**				
• Headache	38	76.0	12	24.0
• Chest pain and shoulder	28	56.0	22	44.0
• Weight loss and loss of appetite	33	66.0	17	34.0
• General weakness	40	80.0	10	20.0
• Hypertension	41	82.0	9	18.0
• Lower limb edema	19	38.0	31	62.0
• Feeling of hypotension	8	16.0	42	84.0
**Lab investigation**				
• Cardiac functions	50	100.0	0	0.0
• CBC	50	100.0	0	0.0
• Kidney function	50	100.0	0	0.0
• Liver functions	50	100.0	0	0.0
• Blood glucose	46	92.0	4	8.0
• ESR – Clotting test	50	100.0	0	0.0
• Urine analysis	22	44.0	28	56.0
• CRP	41	82.0	9	18.0
• Rheumatoid factor	33	66.0	17	34.0
**Radiological investigations**				
• ECG	50	100.0	0	0.0
• Echo	50	100.0	0	0.0
• CT chest	32	64.0	18	36.0
• Other CT	19	38.0	31	62.0
• MRI	28	56.0	22	44.0
• Chest X- ray	39	78.0	11	22.0

Regarding the patients symptoms and signs suffering, lab investigation and radiological investigations, Table 3 showed that 80% suffered from general weakness and 82% had hypertension. 76% had a headache. 100% of the patients studied did cardiac functions, CBC, liver and kidney functions as lab investigations, while 100% did ECG, and Echo. While only 56% and 78% of patients did MRI and chest X- ray respectively.

#### Part III: Patient knowledge regarding foot reflexology for CABG surgery patients

**Table 4:** Number and percentage distribution of the patients studied according to their knowledge regarding foot reflexology for CABG surgery patients (Pre intervention and post intervention) (*N* = 50)

Items	Pre intervention		Post intervention		x^2^	*P*-value
	Yes	%	Yes	%		
Knowledge regarding concept of CABG	21	42%	39	78%	13.365	< 0.001**
Knowledge regarding post CABG treatment and intervention	18	36%	44	88%	28.406	< 0.001**
Knowledge regarding concept of reflexology	11	22%	43	86%	40.812	< 0.001**
Knowledge regarding the concept of importance of reflexology	10	20%	47	94%	55.296	< 0.001**
Knowledge regarding steps of foot reflexology	6	12%	45	90%	60.256	< 0.001**
Total	13	26%	44	88%	38.816	< 0.001**

Using: x^2^: Chi-square test for Number (%), when appropriate p-value > 0.05 is insignificant; *p-value < 0.05 is significant; **p-value < 0.001 is highly significant

Table 4 revealed that there was a statistically significant improvement in knowledge about concept of CABG and post CABG treatment and intervention in post-intervention compared to pre-intervention, where *x*^*2 (*^13.365 and 28.406 respectively) with P-value of *p* < 0.001. While the total knowledge which was (x^*2*^ 38.816) with a P-value of *p* < 0.001.

#### Part IV: Visual analog scale (VAS)” pain scale”

**Table 5:** Number and percentage distribution of the patients studied according to their VAS (Pre intervention and post intervention) (*N* = 50)

Degree of pain	Pre-intervention		Post - intervention		*x* ^*2*^	p-value
	No.	%	No.	%		
No pain	0	0.0	15	30.0	17.471	< 0.001**
Mild	14	28.0	19	38.0		
Moderate	17	34.0	11	22.0		
Severe	13	26.0	4	8.0		
Very severe	4	8.0	1	2.0		
Worst pain possible	2	4.0	0	0.0		

Using: x^2^: Chi-square test for number (%) or fisher’s exact test, when appropriate

Table 5 showed that there increase of the number of people whose pain level increased, for from mild to moderate in pre- intervention (28%: 34%) respectively, comparing post intervention which were from mild to moderate (38% :22%) respectively. And there was a statistically significant reduction in pain in post-intervention compared to pre-intervention, with a p-value of *p* < 0.001.

**Fig. 1:**
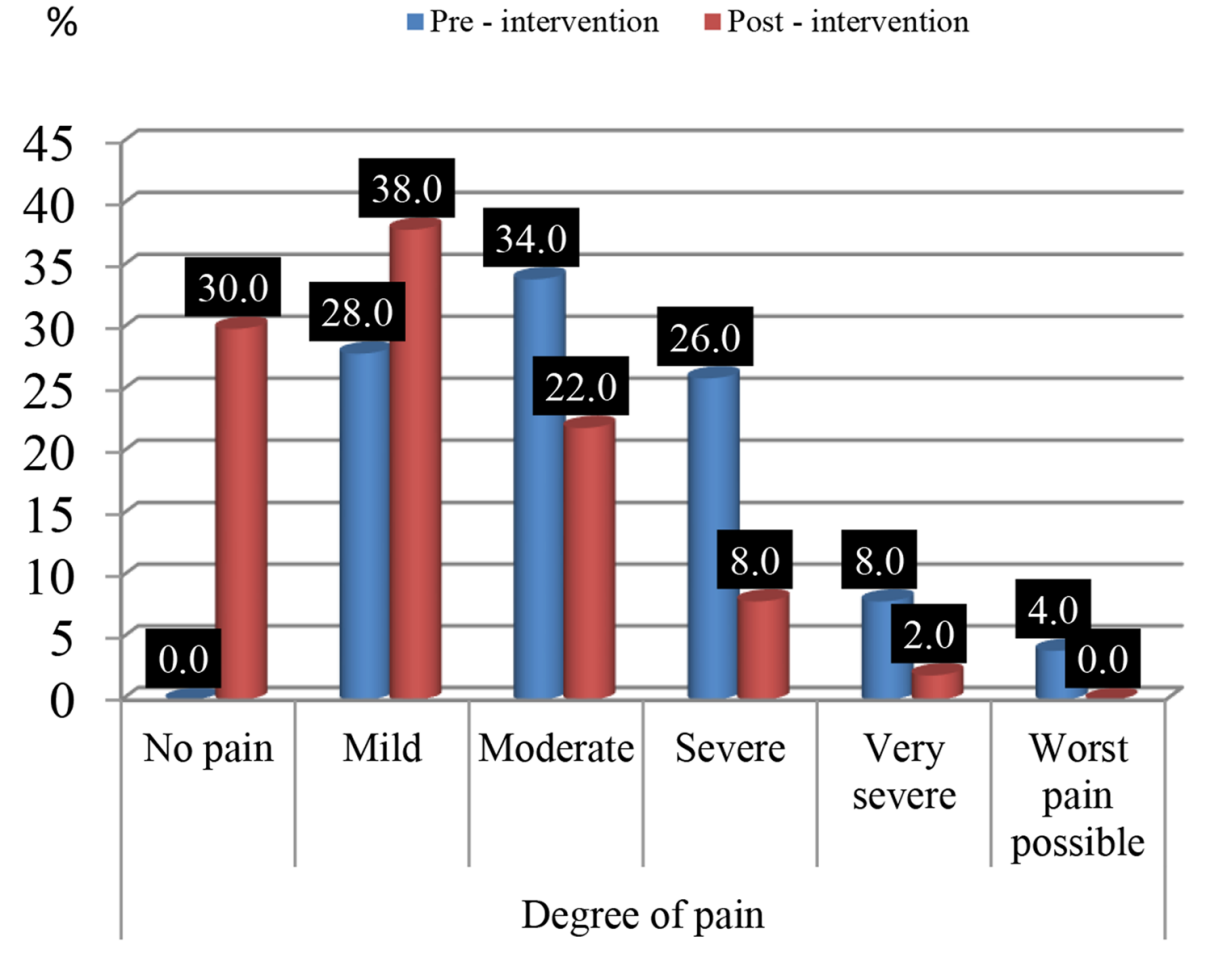
Showed the percentage distribution of the studied patients` degree of pain according to their VAS (Pre intervention and post intervention), which indicated that 38% and 28% had mild pain post and pre intervention respectively, while 30% post interventions had no pain and 4% suffered from worst pain pre interventions

Fig. 1 Percentage distribution of the studied patients` degree of pain according to their VAS (Pre intervention and post intervention)

### Part V: Resilience (The connor–Davidson resilience scale (CD-RISC)

**Table 6:** Mean ± SD distribution of the studied patients according to their resilience (Pre intervention and post intervention) (*N* = 50)

Domains for resilience	Pre-intervention	Post - intervention	Paired Sample t-test	*p*-value
High standards, tenacity, and competence “eight items”	13.88 ± 2.64	18.42 ± 4.05	4.230	< 0.001**
Handling negative emotions, trusting one’s instincts, and perceived benefits of stress “seven items”	12.58 ± 2.39	17.16 ± 3.26	3.816	< 0.001**
The third factor reflects having a positive attitude to change and secure relationships “five items”	8.30 ± 2.41	12.28 ± 2.33	5.567	< 0.001**
The fourth one reflects perceived control “three items”	4.62 ± 0.88	7.38 ± 2.14	3.363	0.009*
The fifth one spirituality “two items”	3.86 ± 0.97	4.96 ± 0.94	2.465	0.012*
Total	43.24 ± 8.22	60.20 ± 15.05	6.293	< 0.001**

Table 6 revealed that there was a statistically significant highest mean value of resilience in post-intervention compared to pre-intervention, with a p-value of *p* < 0.001.

### Correlation between visual analog scale (VAS)” pain scale and total score of resilience

**Table 7:** Correlation between visual analog scale (VAS)” pain scale and total score of resilience (*N* = 50)

Domains for resilience	VAS score			
	Pre-intervention		Post - intervention	
	*r*-value	*p*-value	*r*-value	*p*-value
High standards, tenacity, and competence “eight items”	-0.280	0.751	-0.612	0.001**
Handling negative emotions, trusting one’s instincts, and perceived benefits of stress “seven items”	-0.157	0.509	-0.476	0.005*
The third factor reflects having a positive attitude to change and secure relationships “five items”	-0.161	0.619	-0.377	0.013*
The fourth one reflects perceived control “three items”	-0.100	0.237	-0.334	0.017*
The fifth one spirituality “two items”	-0.111	0.468	-0.783	0.001**
Total resilience	-0.088	0.116	-0.532	0.001**

r-Pearson Correlation Coefficient

Table 7 indicated that there was a statistically significant negative correlation between VAS score and resilience in post-intervention, with a p-value of *p* < 0.05.

## Discussion

For patients undergoing coronary artery bypass graft surgery, foot reflexology dramatically decreased the rise in discomfort and pain following chest tube removal. The study aimed to evaluate the effectiveness of foot reflexology in relieving pain and improving resilience. So, regarding the sociodemographic data, the study showed that most patients were male within the age range of thirty to less than fifty years, with a mean ± SD of 40.20 ± 7.64. This is matched with [18]. Who found in his study that it was identified that more than two-thirds of patients in the test group were males, less than half of patients were between sixty and seventy years old, and more than three-quarters of the patients were married? And more than half of them are smokers and living with smokers at home. From the researchers’ point of view, for older men with coronary artery disease, CABG may be a good option if their surgical risk is manageable.

More than half of the patients have been hospitalized before and have had a family history of heart diseases. This is in line with [19, 20], which refers to coronary artery disease as one of the most pervasive cardiovascular system diseases, and most participants usually have coronary artery disease. On the other hand, less than one-third of them had undergone previous heart surgeries, while the rest had no history of such surgeries. Nearly two-thirds of them take medicine for non-heart disease. This is related to [21], which indicated that more than half of all patients had undergone cardiac valve surgery. And the results showed that in the surgical treatment of cardiac diseases, the two methods were selected for different groups of patients.

Also [22], mentioned that an individual’s risk of coronary artery disease (CAD) and perhaps needing CABG can be raised by a family history of heart disease, particularly premature heart disease (defined as a diagnosis made before the age of 60). Nevertheless, many patients who need CABG do not have a family history of cardiac disease. From the researchers’ point of view, cardiovascular disease has a positive family history, and coronary artery bypass grafting has good long-term results.

As regards the suffering symptoms and signs, more than two-thirds of them had pain, headache, weight loss, and loss of appetite. Nearly all patients did the required laboratory and radiological investigations, such as CBC, cardiac functions and ECG, CT, etc. This is in line with [23], who stated that these kinds of patients usually suffer from physiological parameter disturbances, which include heart rate, respiratory rate, systolic blood pressure, diastolic blood pressure, and oxygen saturation level disturbances.

Also, this is related to [24], who stated that several tests are required prior to coronary artery bypass graft (CABG) surgery to evaluate the patient’s general health and determine the precise ailment that necessitates treatment. ECG, CT, stress testing, cardiac catheterization, heart imaging tests, and a careful examination of the patient’s prescriptions and medical history are all part of these studies. From the researchers’ point of view, all surgical interventions required all laboratory and radiological investigations to ensure that patients are ready for the operations and there are no restrictions.

According to their knowledge regarding foot reflexology for CABG surgery, pre- and post-intervention, the study showed there was a statistically significant improvement in knowledge post-intervention compared to pre-intervention, with a p-value of *p* < 0.001. This is in line with [25], who found a significant relationship between the level of education and quality of life of patients after interventions in components of physical and psychological health, vitality, and social activity.

Also, it is related to [26], who explained that following coronary artery bypass graft (CABG) surgery, patients’ comprehension of their condition and recuperation might be enhanced with organized patient education. Better adherence to postoperative instructions and better health outcomes may result from this increased understanding. From the researchers’ point of view, the educational program for patients undergoing CABG usually enhances their knowledge and understanding of the related issues of the surgery and releases stress and related anxiety.

According to the study, there was a statistically significant decrease in pain after the intervention as compared to before, with a p-value of *p* < 0.001 for the visual analog scale (VAS) “Pain Scale”. This is consistent with [27], who demonstrated that test group patients’ mean VAS scores were lower than those of the control group. It further suggests that the test group patients’ pain levels were lower than those of the control group following the foot reflexology intervention. However, this outcome is consistent with [28], who reported that patients who received foot reflexology experienced greater comfort and less discomfort than those who did not receive reflexology. One non-pharmacological therapeutic option available in postoperative nursing care is reflexology.

Additionally, it is consistent with [9], which mentions that foot reflexology considerably decreased the rise in discomfort and anxiety that patients undergoing coronary artery bypass graft surgery experienced during the removal of their chest tubes. And [29] said that the mean pain scores were considerably reduced in the group who underwent reflexology compared with the placebo group (F = 36.569, *P* = 0.000). Mean anxiety scores were significantly lowered to 60 min for the reflexology group compared with the control group (*P* = 0.000).

In relation to the patient’s resilience, the study showed that there was a statistically significant highest mean value of resilience in post-intervention compared to pre-intervention, with a p-value of *p* < 0.001. As in relation to the mean ± SD of the high standards, tenacity, and competence, the pre-intervention was 13.88 ± 2.64, the post-intervention was 18.42 ± 4.05, and the P-value was < 0.001**. It is consistent with [30]. Who mentioned that resilience is a skill and capacity that can be learned and trained? And his study results showed that people who are more knowledgeable and getting more practice and care usually have more chances to improve their resilience. So, emphasizing and concentrating on patients’ resilience deserves more attention.

Also [31], explored that foot reflexology has been revealed to have a moderating effect on fatigue, stress, anxiety, and cancer. From the researcher’s point of view, by lowering pain, stress, and anxiety, foot reflexology a complementary therapy that applies pressure to areas on the foot may increase a patient’s resilience and improve their general well-being. Research indicates that it can have a good effect on several physiological and psychological markers, which may help people feel more balanced and have better coping skills.

A statistically significant negative connection (p-value *p* < 0.05) was found between the visual analog scale (VAS) pain scale and the overall resilience score in the post-intervention period. This is almost consistent with the findings of [32], who showed a moderate relationship between the VAS and changes in outcomes related to patient satisfaction, particularly for patients having hip arthroscopic surgery. According to the researchers, in patients undergoing Coronary Artery Bypass Grafting (CABG), changes in Visual Analogue Scale (VAS) pain scores and patient satisfaction, along with changes in other Patient Reported Outcomes (PROs) scores, were found to be moderate and statistically significant.

This suggests a connection between patients’ overall satisfaction and other health outcomes and the amount of discomfort they experience after CABG. However, the search results provided don’t go into detail about how pain and resilience are related. Furthermore, it is consistent with [29], who discovered that hand reflexology massage helped patients feel less anxious and in discomfort in the first few days following CABG.

And [33] stated that because vital energy flows from the foot into every part of the body, reflexology can help lessen pain and anxiety. Disease will eventually arise from any obstruction to this flow. It is possible to release energy and remove these obstructions to the passage of the canals in each foot by stimulating specific reflexology spots. By releasing chemicals that resemble morphine, it also deactivates the pain circuits [34]. This conclusion was supported by the findings of [35] who investigated the viability and efficacy of foot reflexology administered following a cardiac operation and found that patients who received it experienced noticeably less pain.

## Conclusion

Particularly for patients who have undergone CABG surgery, foot reflexology is seen as an essential, noninvasive, and readily deployable complementary therapy in which nurses can actively participate. It would lessen the discomfort they endured and speed up their recuperation. For patients with a history of open-heart surgery, it is recommended that nurses be encouraged to carry out this intervention during the postoperative phase. After open heart surgery, foot reflexology significantly improves physiological markers including pain threshold and vital signs.

### Recommendations

Apply a foot reflexology training program for nurses who work in open heart surgery units so they may incorporate it into their regular patient care. Patients should follow foot reflexology training as part of their routine care after open heart surgery. Provide nursing personnel with regular in-service training to aid in the appropriate management of CABG patients to enhance their quality of life and results. Future research should replicate this study with a bigger sample size to generalize.

### Limitations

It’s possible that bias was introduced and that the implementation and results were impacted using self-reported data to gauge patient understanding. Because there was no randomization in the quasi-experimental design, it is difficult to draw conclusions about causality, and baseline variations between the study and control groups could have skewed the results. Furthermore, there is potential for more research into patient perspectives on care given the study’s scant examination of patient experiences beyond satisfaction ratings.

## Supplementary Information

Below is the link to the electronic supplementary material.

Supplementary Material 1

## Data Availability

No datasets were generated or analysed during the current study.

## Abbreviations

CAD: Coronary Artery Disease
CABG: Coronary Artery Bypass Grafting
VAS: Visual Analog Scale
CD-RISC: Connor–Davidson Resilience Scale
