# Supplementary material for: Effect of foot reflexology on relieving pain and improving resilience among patients undergoing coronary artery bypass graft
Source: BMC Nurs. 2025 Sep 10;24:1170. doi: 10.1186/s12912-025-03860-w (PMC12424206; doi:10.1186/s12912-025-03860-w)
Supplement: Supplementary file 1 — Supplementary Material 1 [file 12912_2025_3860_MOESM1_ESM.pdf]

Quesionnair

Effectiveness of Implementing a Nursing Program of Foot Reflexology on Relieving Pain and Improving Resilience: A Framework for Optimizing Nursing Interventions for Patients Undergoing CABG.

- **Please check (√) Infront your suitable answer**

Part one: Demographic data

Item	Yes	No
**Age (years)**		
≤29		
30-≥49		
≥ 50		
**Gender**		
Female		
Male		
**Marital status**		
Single- Widow- Divorced		
Married		
**Level of Education**		
Illiterate		
Read and write		
Highly educated		
**Socioeconomic status:**		
Sufficient		
Not sufficient		
**Treatment cost**		
Free		
Self-cost		
Medical insurance		
**Living area**		
Rural		
Urban		
**Smoking**		
Yes		
No		

Part two: Medical data

Present history		
	Yes	No
**Have previous None heart surgery**		
**Have previous heart surgery**		
**Previous history:**		
• Have family members with heart disease		
• Have family members with heart surgery		

• Entering hospital with other disease		
• Taking medicine for non-heart disease		
**Symptoms and signs suffering**		
• Headache		
• Chest pain and shoulder		
• Weight loss and loss of appetite		
• General weakness		
• Hypertension		
• Lower limb edema		
• Feeling of hypotension		
**Lab investigation**		
• Cardiac functions		
• CBC		
• Kidney function		
• Liver functions		
• Blood glucose		
• ESR – Clotting test		
• Urine analysis		
• CRP		
• Rheumatoid factor		
**Radiological investigations**		
• ECG		
• Echo		
• CT chest		
• Other CT		
• MRI		
• Chest X- ray		

`Part III: Patient knowledge regarding foot reflexology for CABG surgery patients

Items		
	Yes	No
Knowledge regarding concept of CABG		
Knowledge regarding post CABG treatment and intervention		
Knowledge regarding concept of reflexology		
Knowledge regarding the concept of importance of reflexology		
Knowledge regarding steps of foot reflexology		

Part IV: Visual analog scale (VAS)" Pain Scale".

Degree of pain		
	Yes	No
No pain		
Mild		
Moderate		
Severe		
Very severe		
Worst pain possible		

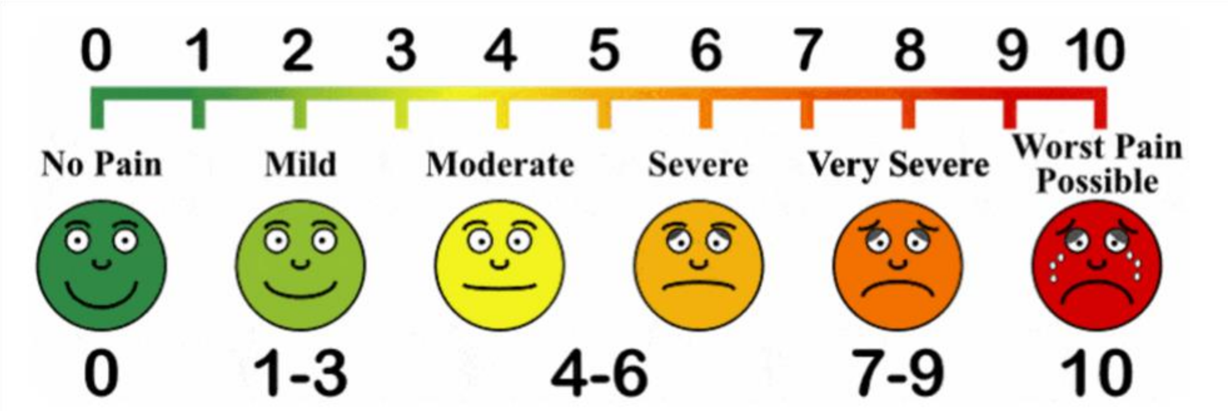

The Connor–Davidson Resilience Scale (CD-RISC).

NO.	Statements
	**High standards, tenacity, and competence "eight items"**
1	Best effort no matter what
2	You can achieve your goals
3	When things look hopeless, I don't give up

NO.	Statements
4	Not easily discouraged by failure
5	Think of self as strong person
6	I like challenges
7	You work to attain your goals
8	Pride in your achievements
**Handling negative emotions, trusting one’s instincts, and perceived benefits of stress "seven items"**	
9	See the humorous side of things
10	Coping with stress strengthens
11	Under pressure, focus and think clearly
12	Prefer to take the lead in problem solving
13	Make unpopular or difficult decisions
14	Can handle unpleasant feelings
15	Have to act on a hunch
**The third factor reflects having a positive attitude to change and secure relationships "five items"**	
16	Able to adapt to change
17	Close and secure relationships
18	Can deal with whatever comes
19	Past success gives confidence for new challenge
20	Tend to bounce back after illness or hardship
**The fourth one reflects perceived control "three items"**	
21	Know where to turn to for help
22	Strong sense of purpose
23	In control of your life
**The fifth one spirituality "two items"**	

NO.	Statements
24	Sometimes fate or God can help
25	Things happen for a reason
